# Supplementary material for: Robust Performance of Culture, qPCR, and Genomic Approaches for Shigella Serotyping in a Pediatric Surveillance Cohort
Source: medRxiv. 2026 Jan 6:2026.01.05.26343460. Preprint. [Version 1] doi: 10.64898/2026.01.05.26343460 (PMC12803388; doi:10.64898/2026.01.05.26343460)
Supplement: Supplement 1 [file media-1.pdf]

Supplementary Table 1. *Shigella* spp. isolates and serotype assignment.

BioSample	Accession	Sample ID	Period	Serogroup	Culture	Isolate TAC	Stool TAC	ShigaPass	Definitive	Genome Completeness	Genome Contamination	MLST
SAMN51230371	SRR35310157	014581	10-Aug-22	flexneri	2a	2b	Negative	2b	2b	99.39	1.1	ST-245
SAMN51230372	SRR35310156	014585	12-Aug-22	flexneri	2a	2a	2a	2a	2a	99.35	0.26	ST-6200
SAMN51230373	SRR35310145	014589	2-Sep-22	flexneri	4a	4a	Negative	4av	4a	98.66	0.53	ST-630
SAMN51230374	SRR35310134	014593	11-Oct-22	flexneri	4a	Not typed	4b	Yv	Yv	98.76	1.05	ST-630
SAMN51230375	SRR35310119	000840	13-Oct-22	flexneri	4a	Not typed	4b	NA	NA			
SAMN51230376	SRR35310108	014601	13-Oct-22	flexneri	4a	Not typed	4b	Yv	Yv	99.30	0.57	ST-630
SAMN51230377	SRR35310125	014605	17-Oct-22	sonnei	sonnei	sonnei	sonnei	sonnei	sonnei	99.60	0.58	ST-152
SAMN51230378	SRR35310124	014609	21-Oct-22	sonnei	sonnei	sonnei	sonnei	sonnei	sonnei	99.57	0.11	ST-152
SAMN51230379	SRR35310123	014613	25-Oct-22	sonnei	sonnei	sonnei	sonnei	sonnei	sonnei	98.36	1.15	ST-152
SAMN51230380	SRR35310122	001625	9-Nov-22	flexneri	2a	2a	2a	NA	NA			
SAMN51230381	SRR35310155	014621	8-Nov-22	flexneri	2a	2a	2a	2a	2a	99.38	0.26	ST-6200
SAMN51230382	SRR35310154	014625	10-Nov-22	flexneri	2a	2a	Not typed	2a	2a	99.41	0.27	ST-6200
SAMN51230383	SRR35310153	001671	10-Nov-22	flexneri	2a	2a	Negative	NA	NA			
SAMN51230384	SRR35310152	001820	17-Nov-22	sonnei	sonnei	sonnei	sonnei	sonnei	sonnei	99.60	0.11	ST-152
SAMN51230385	SRR35310151	002171	25-Nov-22	sonnei	sonnei	sonnei	sonnei	sonnei	sonnei	99.60	0.58	ST-152
SAMN51230386	SRR35310150	002174	25-Nov-22	sonnei	sonnei	sonnei	sonnei	sonnei	sonnei	99.60	0.11	ST-152
SAMN51230387	SRR35310149	014633	25-Nov-22	flexneri	4a	Not typed	Not typed	Yv	Yv	98.39	1.18	ST-630
SAMN51230388	SRR35310148	002182	28-Nov-22	sonnei	sonnei	sonnei	sonnei	sonnei	sonnei	99.60	0.58	ST-152
SAMN51230389	SRR35310147	002187	28-Nov-22	flexneri	4a	Not typed	Not typed	Yv	Yv	99.27	0.57	ST-630
SAMN51230391	SRR35310144	002370	6-Dec-22	flexneri	2b	2b	2b	2b	2b	99.20	0.94	ST-245
SAMN51230392	SRR35310143	002478	14-Dec-22	flexneri	2a	2a	2a	2a	2a	99.35	0.26	ST-6200
SAMN51230393	SRR35310142	002579	21-Dec-22	flexneri	2a	2a	2a	2a	2a	99.43	0.26	ST-6200
SAMN51230394	SRR35310141	002624	23-Dec-22	flexneri	2a	2a	2a	2a	2a	99.40	0.26	ST-6200
SAMN51230395	SRR35310140	002857	9-Jan-23	flexneri	2b	2b	2b	2b	2b	99.39	0.94	ST-245
SAMN51230396	SRR35310139	014641	24-Jan-23	flexneri	2b	2b	2b	2b	2b	99.39	0.94	ST-245
SAMN51230397	SRR35310138	014645	1-Feb-23	flexneri	2a	2a	2a	2a	2a	99.70	0.26	ST-245
SAMN51230398	SRR35310137	014649	6-Feb-23	flexneri	2a	2a	2a	2a	2a	99.70	0.26	ST-245
SAMN51230399	SRR35310136	014657	6-Feb-23	flexneri	2b	2b	2b	2b	2b	99.39	0.94	ST-245
SAMN51230400	SRR35310135	014661	7-Feb-23	flexneri	2a	2a	2a	2a	2a	99.48	0.57	ST-245
SAMN51230401	SRR35310133	014665	8-Feb-23	flexneri	2a	2a	2a	2a	2a	99.70	0.66	ST-245
SAMN51230402	SRR35310132	014669	13-Feb-23	flexneri	2a	2a	2a	2a	2a	99.70	0.26	ST-245
SAMN51230403	SRR35310131	014673	13-Feb-23	flexneri	2a	2a	2a	2a	2a	99.69	0.26	ST-245
SAMN51230404	SRR35310130	014677	14-Feb-23	flexneri	2a	2a	2a	2a	2a	99.20	0.26	ST-245
SAMN51230405	SRR35310129	014681	16-Feb-23	flexneri	1a	1b	1b	1b	1a	99.36	0.31	ST-245

SAMN51230406	SRR35310128	014685	22-Feb-23	flexneri	2b	2b	2b	2b	2b	99.39	0.94	ST-245
SAMN51230407	SRR35310127	014689	24-Feb-23	flexneri	2b	2b	2b	2b	2b	99.39	0.94	ST-245
SAMN51230408	SRR35310126	014693	27-Feb-23	flexneri	2b	2b	2b	2b	2b	98.80	2.77	ST-245
SAMN51230409	SRR35310121	014697	2-Mar-23	flexneri	4a	Not typed	Not typed	Y	Y	99.18	1.08	ST-630
SAMN51230410	SRR35310120	014701	2-Mar-23	sonnei	sonnei	sonnei	sonnei	sonnei	sonnei	99.49	0.17	ST-152
SAMN51230411	SRR35310118	014705	2-Mar-23	sonnei	sonnei	sonnei	sonnei	sonnei	sonnei	99.29	0.11	ST-152
SAMN51230412	SRR35310117	014709	16-Mar-23	flexneri	3a	3a	3a	3a	3a	98.98	0.39	ST-628
SAMN51230413	SRR35310116	005005	29-Mar-23	flexneri	1a	1b	1b	1b	1a	99.20	1.03	ST-245
SAMN51230414	SRR35310115	014717	31-Mar-23	flexneri	2a	2a	2a	2a	2a	99.75	0.26	ST-245
SAMN51230415	SRR35310114	014721	31-Mar-23	flexneri	2b	2b	2b	2b	2b	99.39	1.01	ST-245
SAMN51230416	SRR35310113	005035	5-Apr-23	flexneri	1a	1b	1b	1b	1a	99.33	0.31	ST-245
SAMN51230417	SRR35310112	014729	10-Apr-23	flexneri	2a	2a	2a	2a	2a	99.73	0.87	ST-6200
SAMN51230418	SRR35310111	014733	12-Apr-23	sonnei	sonnei	sonnei	sonnei	sonnei	sonnei	99.57	0.29	ST-152
SAMN51230419	SRR35310110	006007	19-Apr-23	sonnei	sonnei	sonnei	sonnei	sonnei	sonnei	94.67	2.96	ST-152
SAMN51230420	SRR35310109	014741	19-Apr-23	flexneri	1a	1b	1b	1b	1a	99.39	0.31	ST-245
SAMN51230421	SRR35310107	014745	20-Apr-23	flexneri	2b	2b	2b	2b	2b	99.05	1.26	ST-245
SAMN51230422	SRR35310106	014749	2-May-23	flexneri	2a	2a	Negative	2a	2a	99.39	0.26	ST-6200
SAMN51231836	SRR35312069	006335	12-May-23	sonnei	sonnei	sonnei	sonnei	sonnei	sonnei	99.44	0.11	ST-152
SAMN51231837	SRR35312068	006545	22-May-23	flexneri	1a	1a	1a	1a	1a	99.07	0.21	ST-1024
SAMN51231838	SRR35312057	006550	22-May-23	sonnei	sonnei	sonnei	sonnei	sonnei	sonnei	99.60	0.11	ST-152
SAMN51231839	SRR35312030	006735	30-May-23	sonnei	sonnei	sonnei	sonnei	sonnei	sonnei	99.60	0.30	ST-152
SAMN51231840	SRR35312051	006825	31-May-23	flexneri	4a	Not typed	Not typed	Yv	Yv	99.11	5.87	ST-630
SAMN51231841	SRR35312040	006865	31-May-23	flexneri	2b	2b	2b	2b	2b	99.39	0.94	ST-245
SAMN51231842	SRR35312013	006961	7-Jun-23	flexneri	4a	Not typed	Not typed	Yv	Yv	99.30	0.86	ST-630
SAMN51231843	SRR35312009	007375	4-Jul-23	flexneri	2a	2a	2a	2a	2a	99.43	0.26	ST-6200
SAMN51231844	SRR35312008	007379	4-Jul-23	sonnei	sonnei	sonnei	sonnei	sonnei	sonnei	99.44	0.69	ST-152
SAMN51231845	SRR35312007	007591	4-Jul-23	flexneri	2b	2b	2b	2b	2b	99.39	1.05	ST-245
SAMN51231846	SRR35312067	007841	25-Jul-23	flexneri	4a	4a	4a	4av	4a	98.95	0.58	ST-630
SAMN51231847	SRR35312066	007879	24-Jul-23	flexneri	2a	2a	2a	2a	2a	99.70	1.19	ST-245
SAMN51231848	SRR35312065	007883	31-Jul-23	flexneri	2a	2a	2a	2a	2a	99.39	0.68	ST-6200
SAMN51231849	SRR35312064	007901	24-Jul-23	flexneri	1a	1b	1b	1b	1a	99.39	0.41	ST-245
SAMN51231850	SRR35312063	014771	1-Aug-23	flexneri	1a	1b	1b	1b	1a	99.32	0.67	ST-245
SAMN51231851	SRR35312062	014775	15-Aug-23	sonnei	sonnei	sonnei	sonnei	sonnei	sonnei	99.60	0.11	ST-152
SAMN51231852	SRR35312061	014779	17-Aug-23	sonnei	sonnei	sonnei	sonnei	sonnei	sonnei	99.53	0.32	ST-152
SAMN51231853	SRR35312060	014783	24-Aug-23	flexneri	1a	1a	1a	1a	1a	99.23	0.29	ST-1024
SAMN51231854	SRR35312059	014787	24-Aug-23	flexneri	2a	2a	2a	2a	2a	99.59	0.26	ST-245
SAMN51231855	SRR35312058	014791	6-Sep-23	flexneri	2a	2a	2a	2a	2a	99.70	0.26	ST-6200

SAMN51231856	SRR35312056	008524	8-Sep-23	sonnei	sonnei	sonnei	sonnei	sonnei	sonnei	99.60	0.11	ST-152
SAMN51231857	SRR35312055	014795	19-Sep-23	flexneri	2a	2a	2a	2a	2a	99.00	0.36	ST-6200
SAMN51231858	SRR35312054	008864	26-Sep-23	flexneri	Y	Not typed	Not typed	Y	Y	99.27	0.00	ST-630
SAMN51231859	SRR35312037	014803	26-Sep-23	flexneri	2a	2a	2a	2a	2a	99.75	0.28	ST-6200
SAMN51231860	SRR35312036	009076	6-Oct-23	flexneri	2a	2a	2a	2a	2a	99.43	0.31	ST-6200
SAMN51231861	SRR35312035	009191	13-Oct-23	sonnei	sonnei	sonnei	sonnei	sonnei	sonnei	99.60	0.11	ST-152
SAMN51231862	SRR35312034	014807	16-Oct-23	sonnei	sonnei	sonnei	sonnei	sonnei	sonnei	99.60	0.58	ST-152
SAMN51231863	SRR35312033	009554	30-Oct-23	flexneri	3a	3a	3a	3a	3a	99.30	0.32	ST-628
SAMN51231864	SRR35312032	009922	21-Nov-23	flexneri	1b	1b	Negative	1b	1b	99.39	0.31	ST-245
SAMN51231865	SRR35312031	009955	22-Nov-23	flexneri	2a	2a	2a	2a	2a	99.70	0.26	ST-6200
SAMN51231866	SRR35312029	010178	1-Dec-23	sonnei	sonnei	sonnei	sonnei	sonnei	sonnei	99.60	0.11	ST-152
SAMN51231867	SRR35312028	010204	4-Dec-23	flexneri	2a	NA	2a	2a	2a	99.70	0.29	ST-17070
SAMN51231868	SRR35312027	010273	7-Dec-23	sonnei	sonnei	sonnei	sonnei	sonnei	sonnei	99.60	0.23	ST-152
SAMN51231869	SRR35312026	010449	18-Dec-23	sonnei	sonnei	sonnei	sonnei	sonnei	sonnei	99.60	0.26	ST-152
SAMN51231870	SRR35312025	010586	28-Dec-23	flexneri	3a	3a	3a	3a	3a	99.30	0.36	ST-628
SAMN51231871	SRR35312024	011018	16-Jan-24	flexneri	2a	2a	2a	2a	2a	99.39	0.61	ST-6200
SAMN51231872	SRR35312023	011022	16-Jan-24	sonnei	sonnei	sonnei	sonnei	sonnei	sonnei	99.60	0.14	ST-152
SAMN51231873	SRR35312022	011141	18-Jan-24	sonnei	sonnei	sonnei	sonnei	sonnei	sonnei	99.24	0.11	ST-152
SAMN51231874	SRR35312053	011149	18-Jan-24	flexneri	4a	Not typed	Not typed	Yv	Yv	99.30	0.66	ST-630
SAMN51231875	SRR35312052	011206	19-Jan-24	flexneri	2a	2a	2a	2a	2a	99.39	0.26	ST-6200
SAMN51231876	SRR35312050	011269	23-Jan-24	flexneri	2a	2a	2a	2a	2a	99.70	0.29	ST-6200
SAMN51231877	SRR35312049	011633	12-Feb-24	flexneri	1b	NA	1b	1b	1b	99.43	0.26	ST-245
SAMN51231878	SRR35312048	011799	21-Feb-24	flexneri	2a	2a	2a	2a	2a	99.39	0.26	ST-6200
SAMN51231879	SRR35312047	011803	21-Feb-24	flexneri	Y	1a	1a	1a	Y	99.39	0.31	ST-245
SAMN51231880	SRR35312046	011807	21-Feb-24	flexneri	2a	2a	2a	2a	2a	99.39	0.26	ST-6200
SAMN51231881	SRR35312045	012095	27-Feb-24	flexneri	3a	3a	3a	3a	3a	99.41	0.27	ST-245
SAMN51231882	SRR35312044	012103	27-Feb-24	flexneri	1b	1b	3a	3a	1b	99.41	0.27	ST-245
SAMN51231883	SRR35312043	012111	27-Feb-24	flexneri	2a	2a	2a	2a	2a	99.11	0.51	ST-6200
SAMN51231884	SRR35312042	012298	20-Mar-24	flexneri	1b	1b	1b	1b	1b	99.07	0.37	ST-245
SAMN51231885	SRR35312041	012460	25-Mar-24	flexneri	2a	2a	2a	2a	2a	99.43	0.29	ST-6200
SAMN51231886	SRR35312039	012464	27-Mar-24	flexneri	3a	3a	3a	3a	3a	99.33	0.32	ST-628
SAMN51231887	SRR35312038	012468	2-Apr-24	flexneri	2a	2a	2a	2a	2a	99.70	0.26	ST-6200
SAMN51231888	SRR35312021	012476	4-Apr-24	flexneri	1a	NA	Not typed	1a	1a	99.39	0.31	ST-245
SAMN51231889	SRR35312020	012751	15-Apr-24	flexneri	2a	NA	2a	2a	2a	99.35	0.44	ST-6200
SAMN51231890	SRR35312019	012895	24-Apr-24	sonnei	sonnei	sonnei	sonnei	sonnei	sonnei	99.60	0.43	ST-152
SAMN51231891	SRR35312018	012958	2-May-24	sonnei	sonnei	sonnei	sonnei	sonnei	sonnei	99.49	0.58	ST-152
SAMN51231892	SRR35312017	013047	8-May-24	sonnei	sonnei	sonnei	sonnei	sonnei	sonnei	99.60	0.29	ST-152

SAMN51231893	SRR35312016	013260	15-May-24	sonnei	sonnei	sonnei	sonnei	sonnei	sonnei	99.60	0.11	ST-152
SAMN51231894	SRR35312015	013341	18-May-24	flexneri	2a	2a	2a	2a	2a	99.70	0.26	ST-6200
SAMN51231895	SRR35312014	014163	15-Jul-24	sonnei	sonnei	sonnei	Negative	sonnei	sonnei	99.60	0.11	ST-152
SAMN51231896	SRR35312012	014167	17-Jul-24	sonnei	sonnei	sonnei	sonnei	sonnei	sonnei	99.60	0.11	ST-152
SAMN51231897	SRR35312011	014349	31-Jul-24	sonnei	sonnei	sonnei	sonnei	sonnei	sonnei	99.60	0.11	ST-152
SAMN51231898	SRR35312010	014403	7-Aug-24	sonnei	sonnei	sonnei	sonnei	sonnei	sonnei	99.58	0.12	ST-152

Supplementary Table 2. Stool based qPCR serotype assignment.

PID	Serotype
6100216	S.sonnei
6100225	S.sonnei
6100305	S.flexneri 2a
6100331	Untyped
6100393	S.flexneri 2a
6100474	Untyped
6100495	S.sonnei
6100572	S.flexneri 1b
6100585	S.flexneri 2a
6100609	S.sonnei
6100611	S.sonnei
6100653	S.flexneri 2a
6100712	S.sonnei
6100773	S.flexneri 2a
6100845	S.sonnei
6100850	Untyped
6100861	S.sonnei
6100971	S.flexneri 2a
6101001	S.flexneri 1b
6101064	S.sonnei
6200092	S.flexneri 2a
6200136	S.flexneri 4b
6200160	S.flexneri 4b
6200212	S.sonnei
6200235	Untyped
6200321	S.flexneri 2a
6200363	Untyped
6200434	S.sonnei
6200465	Untyped
6200490	S.flexneri 2b
6200532	S.flexneri 2a
6200711	S.flexneri 2a
6200905	S.flexneri 3a
6200910	S.sonnei
6200968	S.flexneri 1b

6201011	S.sonnei
6201027	S.flexneri 2b
6201075	S.sonnei
6201121	S.flexneri 1a
6201139	S.flexneri 1a
6201142	S.sonnei
6201188	S.sonnei
6201195	Untyped
6201214	Untyped
6201238	Untyped
6201277	S.sonnei
6201371	S.flexneri 1b
6201405	S.flexneri 1b
6201422	S.sonnei
6201446	S.sonnei
6201468	S.flexneri 2a
6201479	S.flexneri 2a
6201493	S.flexneri 1a
6201529	S.flexneri 2a
6201565	Untyped
6201635	S.flexneri 2a
6201699	Untyped
6201702	Untyped
6201725	Untyped
6201778	Untyped
6201949	Untyped
6201960	S.sonnei
6201972	S.sonnei
6201985	S.sonnei
6201997	S.sonnei
6202074	S.sonnei
6202095	S.flexneri 2a
6202182	Untyped
6202267	Untyped
6202564	S.sonnei
6202607	S.sonnei
6300154	S.flexneri 4b

6300219	S.flexneri 4b
6300237	S.flexneri 2a
6300255	S.sonnei
6300262	S.sonnei
6300336	S.sonnei
6300397	S.flexneri 2a
6300459	Untyped
6300471	S.sonnei
6300521	Untyped
6300633	Untyped
6300657	Untyped
6300738	Untyped
6300882	Untyped
6300894	S.sonnei
6300918	S.flexneri 2b
6301014	S.flexneri 2a
6301023	S.flexneri 2b
6301092	S.flexneri 2a
6301185	S.flexneri 2b
6301220	S.flexneri 2b
6301356	S.flexneri 4a
6301465	S.flexneri 2a
6301476	Untyped
6301567	S.sonnei
6301580	S.sonnei
6301766	S.sonnei
6301775	S.sonnei
6301782	S.sonnei
6301794	S.sonnei
6301802	S.sonnei
6301816	S.sonnei
6301840	Untyped
6301869	S.flexneri 1b
6301878	Untyped
6301884	S.flexneri 2a
6301922	S.flexneri 2a
6301987	S.sonnei

6302051	S.flexneri 4a
6302085	S.flexneri 1b
6302161	S.sonnei
6302177	S.flexneri 2a
6302189	S.flexneri 2a
6302192	S.flexneri 2a
6302200	S.flexneri 2a
6302217	S.flexneri 2a
6302221	Untyped
6302301	S.flexneri 2a
6302364	Untyped
6302402	Untyped
6302528	S.sonnei
6302543	S.sonnei
6302581	S.sonnei
6302698	S.flexneri 3a
6302710	S.flexneri 2a
6302722	S.sonnei
6302746	Untyped
6302852	S.flexneri 1b
6302890	S.flexneri 1a
6302909	S.flexneri 2a
6302953	S.flexneri 3a
6302966	S.flexneri 3a
6303025	Untyped
6303069	S.flexneri 2a
6303084	S.flexneri 2a
6303198	Untyped
6303318	S.sonnei
6303352	S.flexneri 2a
6303408	S.flexneri 2a
6303692	S.sonnei
6303728	S.sonnei
6400133	S.sonnei
6400169	S.sonnei
6400229	Untyped
6400330	S.flexneri 1b

6400366	S.flexneri 1b
6400401	S.flexneri 2a
6400538	S.sonnei
6400545	S.sonnei
6400646	S.flexneri 2a
6400654	S.flexneri 1b
6400668	Untyped
6400687	S.flexneri 2a
6400721	S.sonnei
6400742	S.flexneri 2a
6400808	Untyped
6400919	S.flexneri 3a
6401088	S.sonnei
6401194	S.flexneri 2a
6500027	S.flexneri 2a
6500142	S.flexneri 4a
6500174	Untyped
6500292	S.flexneri 2a
6500332	S.sonnei
6500380	Untyped
6500405	S.sonnei
6500410	S.sonnei
6500446	Untyped
6500493	S.flexneri 2a
6500583	S.flexneri 2b
6500702	S.flexneri 2a
6500716	S.flexneri 2a
6500740	S.flexneri 2a
6500769	S.flexneri 2a
6500784	S.sonnei
6500791	S.flexneri 2a
6500896	S.flexneri 2b
6500908	S.flexneri 1b
6500913	S.sonnei
6500985	S.sonnei
6501040	S.sonnei
6501057	S.flexneri 1a

6501078	S.flexneri 2b
6501084	Untyped
6501091	S.flexneri 2b
6501103	Untyped
6501115	Untyped
6501126	Untyped
6501159	Untyped
6501209	S.flexneri 2b
6501227	Untyped
6501318	S.flexneri 1b
6501341	S.sonnei
6501352	S.flexneri 1a
6501365	S.flexneri 2a
6501376	S.flexneri 2a
6501424	Untyped
6501522	S.sonnei
6501645	S.sonnei
6501704	S.flexneri 3a
6501728	S.flexneri 2a
6501743	S.sonnei
6501755	S.sonnei
6501762	S.flexneri 2a
6501770	S.flexneri 2a
6501858	S.flexneri 1b
6501886	S.flexneri 2a
6501939	S.flexneri 2a
6501988	Untyped
6502003	S.flexneri 3a
6502015	S.flexneri 2a
6502098	S.sonnei
6502290	S.sonnei
